# Association between serum IgG level and clinical course in primary sclerosing cholangitis

**DOI:** 10.1186/s12876-019-1075-0

**Published:** 2019-08-27

**Authors:** Theresa Hippchen, Peter Sauer, Benjamin Göppert, Peter Schirmacher, Daniel Nils Gotthardt, Karl-Heinz Weiss, Wolfgang Stremmel, Christian Rupp

**Affiliations:** 1 Department of Internal Medicine IV, Heidelberg University Hospital, Im Neuenheimer Feld 410, 69120 Heidelberg, Germany; 2 Institute of Pathology, University of Heidelberg, Im Neuenheimer Feld 224, 69120 Heidelberg, Germany

**Keywords:** Primary sclerosing cholangitis, Hypergammaglobulinaemia, Autoimmune hepatitis, Cholestatic liver disease

## Abstract

**Background:**

Primary sclerosing cholangitis is a chronic cholestatic liver disease. The pathomechanism is still not fully understood, but there is evidence that immune-mediated processes may contribute to disease progression.

**Methods:**

We studied the prognostic relevance of serum immunoglobulin G (IgG) elevated above the upper limit of normal as a marker for immune activation at initial diagnosis and its influence on transplantation-free survival in a well-defined cohort of PSC patients.

**Results:**

The final study cohort comprises of 148 PSC patients. Elevated IgG levels were found in 66 patients (44.6%). Apart from their younger age at first diagnosis, there was no significant difference between patients with or without elevated IgG levels. The presence of a concomitant inflammatory bowel disease, an autoimmune hepatitis or immunosuppressive medication was equally distributed between both groups. Patients with elevated IgG levels reached the combined endpoint (34 (59.6%) vs. 23 (40.4%); *p* = 0.004) significantly more often and had reduced transplantation-free survival (Log-rank: 24.0 (10.2–37.9) vs. 14.0 (8.5–19.5); *p* < 0.05). Cox regression analysis including age, gender, presence of IBD, presence of dominant stricture (DS), Mayo Risk Score (MRS), immunosuppression, biochemical response to UDCA and elevated IgG-levels confirmed MRS (*p* = 0.03), DS (*p* = 0.04), biochemical response (p = 0.04) and elevated IgG level (p = 0.04) as independent risk factors for reduced transplantation-free survival.

**Conclusion:**

We identified elevated serum IgG levels at first diagnosis as an independent risk factor for reduced transplant free-survival in patients with PSC.

## Introduction

Primary sclerosing cholangitis (PSC) is a chronic cholestatic liver disease characterised by progressive sclerosis and destruction of the biliary system [1]. It leads to a liver cirrhosis and a subsequent cholestatic liver failure with the need for orthotopic liver transplantation (OLT) with a median transplantation-free survival between 12 and 18 years and a lifetime occurrence of 15% for cholangiocarcinoma (CCA) [2]. However, the pathogenesis of PSC is not yet well understood. Numerous recent studies have focused on the influence of different genetic risk factors combined with environmental and immunological factors, such as translocation of the gastrointestinal flora to the portal venous system causing chronic inflammation in the biliary system [3–5]. Genetic studies also attribute autoimmune processes a central role in the pathogenesis of PSC [6]. This is explained by the association between PSC and several human leukocyte antigen (HLA) and non-HLA immune regulatory genes, almost all of which have been linked with immune-mediated or autoimmune conditions [7–9]. The assumption that PSC is an immune-mediated inflammatory disease is supported by the frequent association of PSC with inflammatory bowel disease (IBD), as well as the frequent association with other autoimmune diseases like diabetes mellitus type 1 or autoimmune thyroiditis which all worsen the outcome [10, 11]. On the contrary, despite its suspected autoimmune aetiology, PSC does not respond to immunosuppressive drugs. Additionally, it occurs more often with male patients and PSC-specific autoantigens are lacking [12].

In some PSC patients concomitant histological and biochemical features of an autoimmune hepatitis (AIH) can be found [13]. This group was previously diagnosed with the AIH/PSC overlap syndrome or AIH/PSC variant that is typically seen in younger patients [14]. Still, according to the current understanding, this term is rather misleading [15]. It is assumed that it is not a separate entity, but rather belongs to a continuous spectrum of PSC standing out with additional various pronounced features of autoimmune hepatitis, meaning that there is a large heterogeneity of clinical, laboratory and histological manifestations. Immunoglobulin G (IgG) and transaminases might be elevated in these patients. In combination with the histopathology findings the diagnosis of an AIH/PSC overlap syndrome can be made.

Immune-mediated processes seem to play an important role in the pathogenesis and disease activity of PSC. Different autoantibodies such as proteinase-3 antineutrophil cytoplasmatic antibody, anticardiolipin antibodies and anti-GP2 IgA, which are present in patients with PSC, have been assessed [16–18]. However, none of them correlated well with disease progression, nor are they part of the routine work-up in patients with unclassified liver disease.

As PSC especially in its early stages might be mainly autoimmune mediated, we aimed at determining the underlying immunological activity in PSC patients at first diagnosis. For this purpose, serum IgG levels were analysed [19]. We investigated the prognostic relevance of serum IgG levels in a well-defined collective of patients with PSC who were followed up at our tertiary medical centre over the course of up to 28 years.

## Patients and methods

**Fig. 1:**
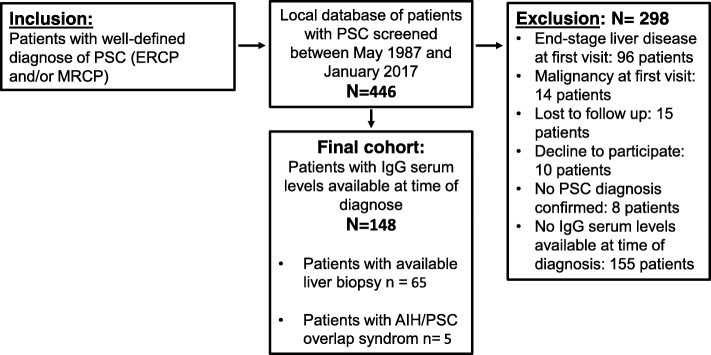
Patient recruitment. Schematic representation of the study design

In this study, we aimed at analysing elevated serum IgG levels above the upper limit of normal as a risk factor associated with reduced transplantation-free survival in patients with PSC. Patients with well-defined PSC who were seen in our in- or outpatient clinics at the University Hospital of Heidelberg between May 1987 and January 2017, were screened from our local database and deemed eligible for inclusion. 446 patients with confirmed PSC were considered for inclusion, 289 of whom were excluded. 10 patients declined to participate in the study, 96 patients were evaluated for liver transplantation due to advanced-stage PSC at first visit (laboratory and/ or clinical signs of compensated or decompensated liver cirrhosis or symptoms of hepatic encephalopathy, presence of ascites, episode of oesophagus variceal bleeding), and 14 patients had malignancies at the first visit (11 cholangiocarcinoma, 1 hepatocellular carcinoma, 2 colorectal carcinoma). Fifteen patients were lost to follow-up and were not considered in the final evaluation. In 8 patients, the diagnosis of PSC was not confirmed. In 155 patients no serum IgG level at first diagnosis of PSC was available. In total, 148 patients had serum IgG levels at first diagnosis available, hence were included in the final study cohort and all of them were followed up until June 2017 (Fig. 1). The combined primary endpoint was defined as OLT or death.

The diagnosis of PSC was established on the basis of typical endoscopic retrograde cholangiography (ERC) or magnetic resonance cholangiopancreaticography (MRCP) findings, serum alkaline phosphatase (ALP) activity of at least twice the reference range, negative antimitochondrial antibody and the results of liver biopsy consistent with the diagnosis of PSC. Time of initial diagnosis was defined as soon as ERC or magnetic resonance cholangiopancreaticography showed typical changes consistent with the diagnosis PSC. At cholangiography, dominant stricture (DS) are defined as stenoses measuring < 1.5 mm in the common bile duct or < 1.0 mm in the hepatic ducts. In patients without a narrowed common bile duct at the first visit, we performed ERC every other year until 1995. From then onwards, ERC was only performed when ALP or GGT increased by 20% or more. In patients in whom DS were identified balloon dilations of the strictures were performed and repeated in scheduled intervals up to resolution of obstructive cholestasis.

Clinical and laboratory data were obtained at initial diagnosis (±3 months) by chart review. Laboratory data included liver function tests (aspartate aminotransferase (AST), alanine aminotransferase (ALT), prothrombin time) as well as cholestatic markers such as ALP, bilirubin and y-glutamyl-transferase (GGT). In addition, we detected immunological processes with IgG as a marker, and checked for the presence of an autoimmune disease in our cohort. The measurement of IgG serum levels was not part of our routine work-up in patients with unclassified liver disease and depended on the clinician who first evaluated the patient. The baseline characteristics including age, gender, history of IBD or MRS, however did not differ between the screened and the enrolled group (Table 1).

**Table 1:** Comparison between the screened and the final study cohort

	Final study cohort *N* = 148	Screened cohort *N* = 446	*p*-value
Gender [Male %f]	105 (70.9%)	312 (70.0%)	*P* = 0.8^2^
Median age at initial diagnose [in years]	33.5 (26–47)	32 (24–44)	*P* = 0.2^1^
Median time of follow-up [in years]	9 (3–14)	9 (4–14)	*P* = 0.9^1^
Patients diagnosed with AIH/PSC overlap	9 (6.1%)	29 (6.6%)	*P* = 0.8^2^
Presence of dominant stenosis	87 (58.8%)	268 (60.6%)	*P* = 0.7^2^
Presence of IBD	99 (66.9%)	315 (70.9%)	*P* = 0.4^2^
BMI	22.7 (17.1–31.9)	22.7 (17.3–56.7)	*P* = 0.5
Mayo Risk Score (MRS)	−0.2 (−3.1–2.3)	−0.1 (− 2.7–2.8)	*P* = 0.8
OLT	32 (21.6%)	107 (30.4%)	*P* = 0.04^2^
Re-OLT	9 (6.1%)	21 (6%)	*P* = 1.0^2^
Death	37 (25%)	66 (18.8%)	*P* = 0.2^2^
Combined end-point (OLT and death)	57 (38.5%)	144 (40.9%)	*P* = 0.6^2^
CCA	12 (8.1%)	31 (8.8%)	*P* = 0.8^2^

^1^Mann-Witney- U test was applied

^2^Chi2-Test was applied

Abbreviation: *AIH/PSC* = autoimmune hepatitis/primary sclerosing cholangitis, *CCA* = cholangiocarcinoma, *IBD* = Inflammatory bowel disease, *MRS* = Mayo Risk Score*OLT* = orthotopic liver transplantation

IgG serum levels as well as all residual laboratory data were considered for analysis when they had been taken ±3 months from the time of diagnosis. No samples were thawed in retrospect. IgM and IgA serum levels at baseline were only available in a limited number of patients and therefore not included in the final analysis.

All patients included in the study received UDCA. Until 1995, all patients were treated with UDCA (Dr. Falk GmbH, Freiburg, FRG) 750 mg/d (9–15 mg/kg per day). Since cholestasis may lead to reduced absorption of UDCA and increasing evidence has shown that higher doses may be more effective [9, 10], the latter was sequentially increased until 2001: In 1995 the patients were treated with an increased dose of 750–1250 mg/d (14–17 mg/kg per day) and starting from 2001 with 1000–1750 mg/d (18–21 mg/kg per day). UDCA doses were not adjusted on the basis of clinical disease activity and no adverse effects of UDCA were observed. Other medications were not administered for the treatment of PSC.

Originally, a liver biopsy was taken in all patients at entry into the study, independent of conclusive ERC or MRCP findings. Since in PSC liver biopsies are of only limited clinical value, starting from 2001 a liver biopsy was no longer considered a prerequisite for entry into the study. Since 2001, only in case of inconclusive MRCP and ERC findings or suspected PSC/AIH overlap was a liver biopsy performed. All biopsies were read blindly. Altogether in 65 patients the diagnosis was confirmed by biopsy. As histopathological criteria were considered concentric ‘onion skin’ periductal fibrosis, bile duct proliferation, chronic periportal inflammatory change, cholangioectasia and ductopenia. According to the current EASL guideline, AIH/PSC overlap syndrome is difficult to diagnose with a sufficiently high certainty since standard diagnostic criteria are lacking [20]. In clinical practice though, the guideline allows for patients with typical features of PSC in cholangiography or histology alongside with robust biochemical, serological and histological features of AIH to be diagnosed with AIH/PSC overlap syndrome. In order to diagnose the AIH component, we applied the *Simplified Diagnostic Criteria of the International Autoimmune Hepatitis Group* [21]. The score includes several parameters such as antinuclear (ANA) and/or smooth muscle antibodies (SMA), serum IgG levels, and liver histology with evidence of hepatitis and the absence of viral hepatitis. A score of ≥7 defines AIH. AIH was only diagnosed when a liver biopsy had been available.

In the beginning of the study, IgG4-associated cholangitis has not been known yet which explains why it was not initially determined routinely. However, in a number of patients the PSC diagnosis was confirmed by a liver biopsy during this time period, making an IgG4-associated cholangitis very unlikely. Serum IgG4 was determined starting in 2008 in all our patients at least once. In case of elevated serum IgG4 levels a liver biopsy was performed to rule out IgG4-associated cholangitis. In 17 patients IgG4 level were elevated up to 2 x ULN. In all 17 patients a liver biopsy was performed showing no sign of IgG4-related sclerosing cholangitis. Immunoglobulin levels were measured by using the nephelometric measurement technique. Statistical analyses were conducted using SPSS version 21 *(IBM Corp., Armonk, New York, USA*). Data are presented as a median with an interquartile range (IQR) in the case of continuous variables and as numbers with percentages in the case of categorical variables. For qualitative data, significance was tested using the Chi [2]-, Mann-Whitney-U-test and Fisher’s exact test. Correlation between two continuous variables was calculated using Pearson’s correlation coefficient. The transplantation-free survival rate in our cohort was estimated using the Kaplan-Meier product limit estimator. Differences were tested using the log-rank test. To assess the prognostic significance, we included into the multivariate Cox regression model known risk factors like the Mayo Risk Score (MRS), the presence of DS, IBD, response to UDCA treatment according to the Toronto criteria (ALP < 1.67 x ULN after 24 month UDCA), immunosuppression drugs for the treatment of concomitant AIH or IBD and elevated serum IgG-levels. Significance was defined as *p* < 0.05. The study was previously approved by the local ethics committee in Heidelberg (Approval No. S-043/2011) and was conducted in accordance with the Declaration of Helsinki.

## Results

The final study cohort comprised of 148 PSC patients. To exclude selection bias, we compared the screened PSC cohort with the subset of PSC patients with available IgG levels. Both groups showed no statistical difference with regard to the baseline clinical or laboratory characteristics (e.g. gender, age, presence of AIH/PSC overlap or presence of DS) (Table 1). The majority of patients were male (105 patients; 70.9%), and the median age at the time of diagnosis was 33.5 (26.0–47.0) years (Table 2). All but two patients were Caucasians. Patients had a normal liver function test and the median Mayo Risk Score at baseline was − 0.521 (range: − 1.15 – 0.52; “low risk group”) without any difference between both groups. Due to our exclusion criteria no patient had evidence of liver cirrhosis when considered for the study. The presence of cirrhosis was ruled out at the entry into the study histologically or by non-invasive imaging (ultrasound and/ or MRI, e.g. signs of portal hypertension) or laboratory parameters (e.g. thrombocytopenia, hypoalbuminia) in all patients.

**Table 2:** Baseline characteristics of our study cohort

	N (%)	Median (IQR)	Reference values
Gender [Male %]	148	105 (70.9%)	
Median age at initial diagnose [in years]	148	33.5 (26–47)	
Median time of follow-up [in years]	148	9 (3–14)	
Patients diagnosed with AIH/PSC overlap	5 (3.4%)		
Presence of dominate stenosis	87 (58.8%)		
Presence of IBD	99 (66.9%)		
Histopathological proof	65 (43.9%).		
Presence of type I diabetes	4 (2.7%)		
OLT	32 (21.6%)		
Re-OLT	9 (6.1%)		
Death	37 (25%)		
Combined end-point (OLT and death)	57 (38.5%)		
CCA	12 (8.1%)		
Bilirubin [mg/dl]	142	0.8 (0.56–1.6)	─ 1 mg/dl
ALT [IU/l]	145	98.1 (53.5–230.2)	─ 35 IU/l
AST [IU/l]	145	63.5 (32.5–120.5)	─ 37 IU/l
AP [IU/l]	142	265 (151.5–518.0)	40 ─ 130 IU/l
GGT [IU/l]	148	324.0 (151.5–681.5)	6 ─ 26 IU/l
Albumin [g/dl]	137	44.0 (40.0. – 46.0)	30 ─ 50 g/dl
Serum-IgG levels [g/l]	148	14.9 (12.0–20.2)	─ 16 g/l
Mayo risk score	148	−0.2 (−3.1–2.3)	
MELD	142	6 (6–15)	

Abbreviation: *AIH/PSC* = autoimmune hepatitis/primary sclerosing cholangitis, *IBD* = inflammatory bowel disease, *OLT* = orthotopic liver transplantation, *CCA* = cholangiocarcinoma, *ALT* = alanine aminotransferase, *AST* = aspartate aminotransferase, *AP* = alkaline phosphatase, *GGT* = y-glutamyl-transferase, *MELD score* = Model for End-stage Liver Disease

Since liver biopsies are of limited clinical value in PSC, we only obtained histopathological samples in 65 patients (43.9%). Histopathology was compatible with PSC and did not show conflicting disease or any evidence of the presence of a liver cirrhosis. One liver biopsy was inconclusive, but the diagnosis was confirmed by typical endoscopic findings. We found that of 65 patients who had a liver biopsy conducted, 27 patients showed elevated IgG serum levels (41.5%). No significant difference to the group of patients that did not undergo a liver biopsy was found: In this group, 39 out of 83 patients (47%) had elevated IgG serum levels (*p* = 0.5).

**Fig. 2:**
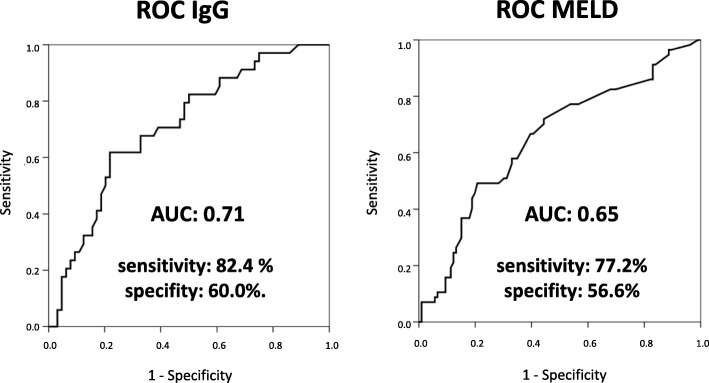
ROC analysis. AUROC analysis indicated that the best cut-off across the entire study cohort for serum IgG was 14.0 g/l, resulting in a sensitivity of 82.4% and specificity of 60.0%. AUROC for MELD score at first diagnosis, was 0.65 (95% CI 0.6–0.7; *p* = 0.002) for MELD at first diagnosis, compared to 0.71 (95% CI 0.6–0.8; *p* < 0.001) for serum IgG level at first diagnosis of PSC

The median time of follow-up was 9 years (range: 3–14). This was defined as the time of diagnosis until the primary end-point or end of the follow-up period was reached. During the study, 25 (16.9%) patients died, 32 (21.6%) underwent an OLT and 91 (61.5%) patients were still under regular follow-up. In total, 99 (66.9%) patients had concomitant IBD and 87 (58.8%) showed DS (Table 2). Amongst the patients with DS, 50 (57.5%) developed the stricture in the course of their disease and 37 (42.5%) presented this at their initial PSC diagnosis. The AIH/PSC overlap syndrome was diagnosed in 5 (3.4%) patients. In 3 (2.0%) patients a small-duct PSC was diagnosed. Mean serum IgG-levels were 14.86 g/l (12.03–20.17) and elevated serum levels (> 16 g/l) were found in 66 patients (44.6%) (Table 2). The results of the ROC analysis indicated that the best cut-off across the entire study cohort for serum IgG was 14.0 g/l, resulting in a sensitivity of 82.4% and specificity of 60.0%. AUROC for MELD score at first diagnosis, was 0.65 (95% CI 0.6–0.7; *p* = 0.002) for MELD at first diagnosis, compared to 0.71 (95% CI 0.6–0.8; *p* < 0.001) for serum IgG level at first diagnosis of PSC (Fig. 2). Patients with elevated IgG levels were significantly younger (30 years [23–39] vs. 40 years [28–50]; *p* = 0.001) than patients with IgG-levels within the normal range (Table 3). Besides this, all other clinical and laboratory characteristics at baseline were comparable. There was no significant correlation between baseline bilirubin, ALP, GGT, ALT or AST and elevated IgG levels, along with known risk factors for PSC like the presence of DS, IBD or MRS at baseline. Positive ANA titer was found in 24 patients (21.4%) and did also not correlate with elevated IgG level. In 103 patients IgG4 serum levels were measured. In 17 patients IgG4 level were elevated up to 2 x ULN. In all 17 patients a liver biopsy was performed showing no sign of IgG4-related sclerosing cholangitis. p-ANCA was measured in 133 patients. In 22 patients p-ANCA was within the normal range. In the other patients p-ANCA was elevated. There was no correlation between p-ANCA titer and IgG levels.

**Table 3:** Subgroup analysis of IgG-levels

	Study cohort	Normal IgG levels (*N* = 82)	Elevated IgG levels (*N* = 66)	*p*-value
Gender [Male %]	105 (70.9%)	55 (67.1%)	50 (75.8%)	0.3^2^
Median age at initial diagnose [years]	33.5 (26–47)	40 (28–50)	30 (23–39)	**0.001** ^**1**^
Presence of IBD [N, %]	99 (66.9%)	50 (50.5%)	49 (49.5%)	0.1^2^
AIH/PSC overlap [N, %]	5 (3.4%)	2 (2.4%)	3 (4.5%)	0.7^3^
Dominant stenosis	87 (58.7%)	48 (55.2%)	39 (44.8%)	0.5^2^
BMI	22.7 (17.1–31.9)	22.2 (17.1–27.1)	23.4 (19.9–31.9)	0.1
Mayo Risk score	−0.521 (−1.15–0.52)	−0.273 (−1.07–0.83)	−0.107 (− 1.02–1.01)	0.4^1^
MELD	6 (6–15)	6 (6–15)	6 (6–14)	0.4
Presents of CCA [N, %]	12 (8.1%)	7 (8.5%)	5 (7.6%)	0.8^2^
Death [N, %]	37 (25%)	15 (18.3%)	22 (33.3%)	**0.04** ^**2**^
OLT [N, %]	32 (21.6%)	13 (15.9%)	19 (29.8%)	0.17^2^
Re-OLT [N, %]	9 (6.1%)	4 (4.9%)	5 (4.6%)	0.5^2^
Combined endpoint (death and OLT)	57 (38.5%)	23 (40.4%)	34 (59.6%)	**0.004** ^**2**^

Table shows differences between the subgroup of patients with normal (N = 82) and elevated IgG-levels (N = 66). Patients with elevated IgG levels were significant younger than patients with IgG levels within the normal range (*p* < 0.005). Patients with elevated IgG levels died more often or reached the combined endpoint (death and OLT) compared to patients that had IgG levels within the normal range (*p* < 0.05 and *p* < 0.005, respectively. There was a trend towards an increased number of orthotopic liver transplantation (OLT) in patients with hypergammaglobulinemia (*p* = 0.057). Bold values indicates significant *P*-values (<0.05)

Abbreviations: *IBD* = inflammatory bowel disease, *AIH/PSC* = autoimmune hepatitis**/**primary sclerosing cholangitis, *MELD score* = Model for End-stage Liver Disease, *CCA* = cholangiocarcinoma, *OLT* = orthotopic liver transplantation

^1^Mann-Witney- U test was applied

^2^Chi2-Test was applied

^3^Fisher’s exact test was applied

Occurrence of dominant strictures or recurrent cholangitis was not influenced by increased serum IgG levels. In total 87 patients (58.8%) developed dominant strictures during follow-up. 39 (40.2%) out of 97 patients had elevated serum IgG levels, whereas 27 patients with elevated serum IgG levels showed no signs of dominant strictures in ERCP (*p* = 0.9). Recurrent episodes of cholangitis were detected in 10 patients. In this group, six patients had elevated serum IgG levels, whereas 51 out of 105 patients had elevated serum IgG levels with no symptoms of cholangitis (*p* = 0.5).

We also studied the presence of autoimmune hepatitis (AIH) depending on IgG levels in our cohort. In total, 3 out of 5 patients with AIH/PSC overlap syndrome and 63 out of 143 patients without AIH/PSC overlap had elevated IgG levels (*p* = 0.7). Other autoimmune diseases, e.g. diabetes mellitus type 1 and Sjogren’s disease, had no influence on IgG levels (p = 0.5). All patients with AIH overlap syndrome received immunosuppression therapy according to the current guidelines [19]. Therapy was initiated with prednisolone monotherapy and then continued with azathioprine and/or low dose prednisolone to maintain remission. Immunosuppression therapy was also administrated in some patients with IBD. 11 patients (2.2. %) received either azathioprine or methotrexate. In 3 patients (3%) IBD was diagnosed and immunosuppression therapy was initiated before PSC was diagnosed. All 3 patients had elevated IgG levels at baseline, while no statistically significant difference to patients without immunosuppression therapy at baseline was found (*p* = 0.1).

Over the study period, 57 patients reached the combined endpoint death and/or OLT. Patients with elevated IgG levels died more often or reached the combined endpoint (death and OLT) compared to patients that had IgG levels within the normal range. [22 patients (33.3%) vs. 15 (18.3%) *p* = 0.036]; 34 (59.6%) vs. 23 (40.4%); *p* = 0.004; respectively]. There was a trend towards an increased number of OLT in patients with elevated IgG levels (*p* = 0.057). (Table 3) 9 out of 32 patients (28.1%) needed a re-liver transplantation. Three patients had transplantation due to a liver failure, three due to a developed chronical rejection, another one suffered from liver abscesses and two patients developed a PSC relapse. Fisher’s exact test does not show significant differences of IgG levels at baseline between patients with a PSC relapse after OLT and patients without a relapse. Due to the small sample size in one group (*n* = 2) statistical tests are probably underpowered and do not allow for interpreting profoundly.

**Fig. 3:**
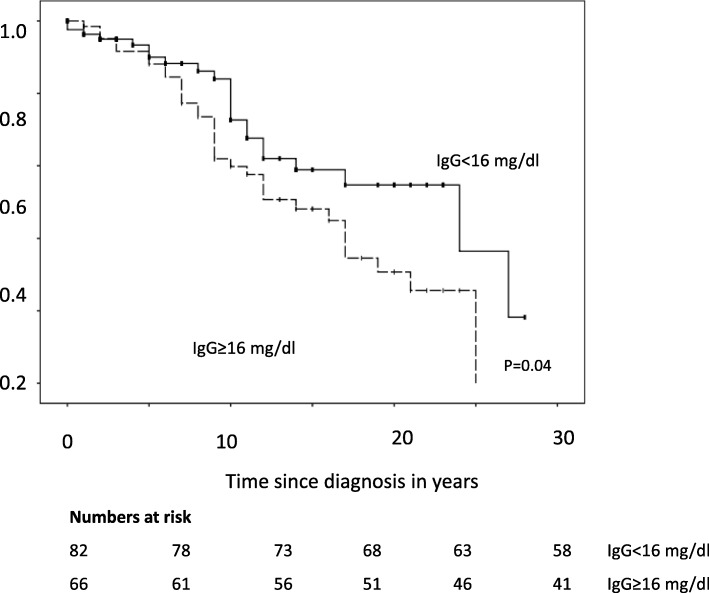
Survival free of liver transplantation. Log-rank test showed shortened median survival in patients with elevated IgG levels compared to patients with IgG levels within the normal range [24.0 (10.2–37.9) vs. 14.0 (8.5–19.5); *p* < 0.05]

In Kaplan-Meier analysis, elevated IgG levels (Log-rank: 24 years [10–38] vs. 14 years [9–20]; *p* = 0.04), and DS (Log-rank: 25 years [14–26] vs. 12 years [9–15]; *p* = 0.009) were associated with shortened transplant-free survival (Fig. 3). In contrast, there was no association regarding IBD or gender (*p* = 0.841; *p* = 0.218 respectively).

In the Cox regression analysis, we included variables that are known risk factors for PSC [1]. We included age at diagnosis, gender, the presence of DS, presence of IBD, MRS, administration of immunosuppression drugs, biochemical response to UDCA treatment according to Toronto criteria and elevated IgG levels. There was a positive correlation between patients with elevated IgG levels and younger age (30 years (23–39) vs. 40 years (28–50); *p* = 0.001). The other variables showed no significant correlation with IgG serum level. In univariate regression analysis, MRS (*p* < 0.005), the presence of DS (*p* = 0.003), biochemical response to UDCA (*p* = 0.01) and elevated IgG-levels (*p* = 0.02) were statistically significant associated with reduced survival. The multivariate cox regression analysis confirmed DS (*p* = 0.04), MRS (*p* = 0.03), biochemical response to UDCA (*p* = 0.04) and elevated IgG levels (p = 0.04) as independent risk factors for reduced transplantation-free survival (Table 4).

**Table 4:** Uni- und multivariate analysis cox regression analyses

	Univariate			Multivariate		
Risk factors	β-estimate	Exp (B) CL 95%	*p*-value	β-estimate	Exp (B) CL 95%	*p*-value
Gender	0.8	0.5–1.4	0.5	1.3	0.5–3.8	0.6
Age of diagnose [years]	1.0	0.9–1.0	0.07	0.9	0.9–1.0	0.8
Presence of IBD	1.7	0.9–3.4	0.1	0.8	0.3–1.9	0.8
Presence of dominant stenosis	2.6	1.4–4.9	**0.003**	3.3	1.1–9.8	**0.04**
Mayo Risk score	1.7	1.3–2.2	**0.001**	1.5	1.0–2.1	**0.03**
Immunosuppression	0.5	0.1–2.1	0.4	0.0	0.0–0.0	1.0
Biochemical response	2.0	1.2–3.4	**0.01**	2.3	1.1–5.4	**0.04**
Elevated Serum IgG-levels	2.4	1.1–5.0	**0.02**	2.4	1.0–5.7	**0.04**

Table shows prospective factors for longer survival until death or liver transplantation. In univariate analysis Mayo Risk Score (MRS), presence of dominant stenosis, biochemical response to UDCA and elevated serum IgG levels were associated with reduced survival. In multivariate analysis, MRS, presence of dominant stenosis, biochemical response to UDCA and elevated serum IgG-levels were independently associated with reduced transplantation-free survival (*p* < 0.05). Bold values indicates significant *P*-values (<0.05)

## Discussion

In this cohort study, we were able to identify elevated serum IgG levels at initial diagnosis as an independent risk factor for reduced transplantation-free survival in patients with PSC. Elevated IgG levels could only be partly explained by the presence of the AIH/PSC overlap syndrome. At baseline, we showed that there is not any difference in clinical baseline characteristics except for younger age in patients with elevated serum IgG levels. After a follow-up period of up to 28 years, PSC patients with elevated serum IgG levels had a markedly reduced transplantation-free survival compared to patients with IgG levels within the normal range at baseline. Reduced transplantation-free survival was caused by the more rapid disease progression seen in patients with elevated serum IgG level. This subgroup of patients might suffer from a more aggressive, immune-driven course of disease. The negative effect of elevated IgG was independent of concomitant DS or IBD.

Elevated immunoglobulin levels in general are associated with autoimmune-mediated or allergic processes. It is assumed that PSC is to some extent an immune-driven process. However, the exact relevance of the association between PSC and immunoglobulins is still poorly understood. Tabibian et al. evaluated the prognostic relevance of IgE levels in a western population of patients with PSC [22]. They did not find an association between IgE levels and survival. Still, the group expressed that elevated IgE levels were linked to significantly higher total IgG and IgG subclasses. The clinical relevance of this finding remained uncertain but supports our results that immunoglobulins play a role in the pathogenesis of PSC. Measurements of IgM, IgA and IgE serum levels were not part of the routine work-up in our cohort and only available in a subgroup of patients. Due to the small sample size and no effect on outcome, the results were not presented in the study.

Elevated IgG serum levels might also be a sign for end-stage liver disease in patients with liver cirrhosis [23]. In order to rule out this possible confounder we excluded patients with liver cirrhosis at first diagnosis of PSC from our study cohort. We found no difference between patients with or without elevated IgG serum levels with regard to disease stage at first presentation. The median Mayo Risk Score of our cohort though was within the “low risk group” at first presentation without any difference between patients with or without elevated IgG levels. This was owed to the fact that patients with end-stage liver disease at first presentation were excluded due to our study protocol. Furthermore, the presence of liver cirrhosis was ruled out in at least a subset of patients with a liver biopsy at entry into the study. We therefore consider it rather unlikely that IgG serum levels were influenced by end-stage liver disease in our cohort, but more likely an expression of stronger reaction of immunity.

We found that younger patients had significantly higher IgG levels and were a group that is at risk of shortened survival for this reason. In contrast to our findings, it has been previously reported that late-onset PSC and increased age in general are associated with poor survival promoted by the increased CCA risk over time [24–26]. However, our data underlines the fact that close surveillance is important in young patients with elevated IgG levels. These patients might suffer from a more immune-driven phenotype of the disease.

Diagnostic criteria for AIH are well established and AIH patients respond to immunosuppressive therapy in the majority of cases. Lüth et al. reported that serum IgG levels correlate well with histological disease activity in patients with established AIH [27]. Several studies have evaluated immunosuppressive regimens in PSC and were not able to provide any evidence for an improvement in laboratory parameters or clinical course [28, 29]. However, in the case of PSC/AIH overlap, immunosuppression may successfully suppress the autoimmune-driven component of the overlap syndrome [14]. Diagnostic criteria for PSC/AIH overlap syndrome usually comprise of both criteria for a separate PSC and AIH. In our cohort 5 (3.4%) patients were diagnosed with PSC/AIH overlap syndrome and treated according to the current guidelines. Interestingly, IgG levels were not statistically different between the AIH/PSC overlap and the PSC-only group. This indicates elevated IgG level not only being a sign for an autoimmune-mediated process but also being likely to point towards a subgroup of PSC patients with a more immunologically-driven pathogenesis. There is one study of Schulze et al., which reported that patients with PSC and elevated IgG levels and liver cirrhosis at baseline are more prone to receive immunosuppression therapy with satisfying therapeutic response [30]. The group of Mendes et al. evaluated the effect of elevated IgG4 levels on prognostically relevant parameters and survival in patients with PSC [23]. The data showed that patients with elevated IgG4 levels had a more severe course of liver disease with shortened transplant-free survival. The authors speculate that these patients might benefit from a treatment with corticosteroids, since IgG4 could be a marker for steroid sensitivity [31].

However, with the available evidence mentioned above and our studies it is unclear whether this subgroup of patients might benefit from immunosuppressive therapy.

Certain limitations affected our study. First, we report about a single centre cohort at a tertiary care centre. Due to the limited effect size of serum IgG level, a validation of our results in an independent replication cohort would be desirable. Second, a systematic IgG measurement was not part of the initial study protocol. Therefore, we could only include a limited subgroup of the initial cohort of PSC patients. Statistical analysis though, did not reveal any significant difference between our initial data and the one of the study cohort. In consequence, we consider the cohort of PSC patients evaluated to be representative. The reliability of the date at initial diagnosis is a further limitation of this study. We do not rule out that we might have missed an AIH/PSC overlap syndrome because in all patients we did neither perform a liver biopsy and nor routinely measured liver-specific autoantibodies. However, our standardised diagnostic, screening, and therapy protocol, including UDCA and endoscopic treatment, should allay this concern of missing an AIH/PSC overlap syndrome. Furthermore, due to our long follow-up period having missed a patient with AIH is rather unlikely.

## Conclusions

In conclusion, we were able to identify elevated IgG levels as an independent risk factor for transplantation-free survival in PSC. This risk marker for a more rapid disease progression might be taken into account for planning individual, risk-adapted screening and surveillance strategies. Despite validation of our results in other PSC cohorts further studies are necessary in order to determine the underlying cause of elevated IgG levels and assess the potential benefit of immunosuppression therapy in selected PSC patients.

## Data Availability

The datasets used and/or analysed during the current study are available from the corresponding author on reasonable request.

## Abbreviations

AIH: Autoimmune hepatitis
ALP: Alkaline phosphatase
ALT: Alanine aminotransferase
ANA: Antinuclear antibodies
AST: Aspartate aminotransferase
CCA: Cholangiocarcinoma
DS: Dominant stricture
ERC: Endoscopic retrograde cholangiography
GGT: y-glutamyl-transferase
HLA: Human leukocyte antigen
IBD: Inflammatory bowel disease
IgG: Immunoglobulin G
MRS: Mayo risk score
OLT: Orthotopic liver transplantation
PSC: Primary sclerosing cholangitis
SMA: Smooth muscle antibodies
UDCA: Ursodeoxycholic acid
